# Virulence profile: Liise-anne Pirofski

**DOI:** 10.1080/21505594.2017.1382277

**Published:** 2017-11-10

**Authors:** Liise-anne Pirofski

**Affiliations:** Medicine, Division of Infectious Diseases, Albert Einstein College of Medicine, Bronx, NY, USA

## Tell us about your early days

I grew up in Northern California – along highway 101. We moved there in 1958, with the baseball Giants as my father used to like to say. I witnessed the growth of the area, transforming from acres and acres of fruit trees to the extension of highway 101 to eventually, the lap of Silicon Valley, albeit after I left the area. I grew up in a home that valued education, discourse, and the liberal arts, with little exposure to science, completely oblivious to STEM. I was an excellent student and was somehow able to convince my school and parents to let me go to college at Berkeley in lieu of my senior year of high school, at age 16.

## Did you have a particular career wish as a child?

I wanted to be a play by play announcer for the San Francisco Giants – in those days – the late 1950s and 1960s – the idea of a female announcer was way beyond anyone's radar screen. Therefore, I spent quite a bit of time searching for a Plan B.

## When did you first get interested in science?

I was always ‘interested’ in science – my mother's heroine was Marie Curie and I was very taken with geology and space travel, the only real exposures I had to science. However, as I guess a ‘typical’ female of that era, I was not pointed in the direction of science during my education. It certainly was not a direction in which any females I knew were steered. When I went to Berkeley, I considered science courses but was very intimidated by everyone who seemed very prepared for and good at it. Instead, I majored in art history and psychology. I am very glad I did – my studies gave me a glimpse into human suffering and disease that has been very helpful to me in caring for patients and ultimately led to my decision to study medicine.

## How did you get interested in science?

My interest in science grew from my experiences in medicine. I think the turning point was internship – at Bellevue in 1982 – taking care of patients with AIDS (before the virus had been identified, when there was no testing, which began in 1985, the year I finished my residency). The patients were sick, dying, and young, we had nothing to offer, did not know what to do, and worst of all – whatever paradigms we had did not explain what was happening to them. Our concepts did not work. Our therapies did not work, even when we knew what we were treating. My frustration, and disbelief that this could be the case after 4 years of medical school and 3 years of residency, led me to go into infectious diseases and then try my hand at science.

## When did you decide to become a scientist?

I took on my fellowship hoping it would give me an opportunity to understand susceptibility to infectious diseases better. When I was a fellow we had a training grant at Einstein that enabled us to do research and I went to work in the lab of Matthew Scharff, on antibodies. I had developed an interest in immunology as a fellow and in Matt's lab I became very interested in antibodies to encapsulated pathogens; that they came from a restricted repertoire and perhaps people who were susceptible to them had a ‘hole in their repertoire’. I still don't know if this is true, but it led to over two decades of fascinating work on antibodies and B cells for me and my group.

## Were there any people who influenced your decision?

Yes. In medical school – Marshall Horwitz – a paediatrician and researcher – my first attending on my paediatrics rotation and the first person I realized was a physician-scientist. I never knew the ‘discipline’ existed. Also, Steve Baum, another mentor in medical school, who was a developer of the MD-PhD program at Einstein. And of course, Matthew Scharff, who took me into his lab even though I had no background in science and – at the time – no clear potential as a scientist. I was lucky enough to get a physician-scientist award (the type of award that preceded the K awards at NIH), which gave me time to develop my career.

## Tell us about your education and experiences at university

College was a terrific experience – I went to Berkeley – one of the greatest universities in the world. As above, I majored in art history and psychology. Although not science – my studies were rigorous, my courses were taught by leaders in their fields, scholars, researchers. One of my psychology professors was in the field of experimental psychology – it was my first introduction to research that leveraged manipulation of conditions in animals to test hypotheses. It was fascinating and in retrospect probably planted a seed that would later blossom as my career as a scientist.

## What do you like most about your work as a physician?

I like people and their stories and I like the opportunity to improve people's health. As an infectious diseases physician, I like that the field is global and we need to be sensitive to culture, history, politics and measures of health near and far.

## What is your position at your institution?

I am chief of the division of infectious diseases at Montefiore/Einstein. It is a large, diverse group of researchers, clinicians and educators. It is a great honor to serve in this position. It has given me an opportunity to mentor the next generation of ID physicians and and scientists.

## What areas or topics does your lab currently focus on?

We are interested in immunity to encapsulated microbes, using pneumococcus and Cryptococcus as examples. Our work is broad-based and ranges from translational studies of human cohorts, to characterization of B cell and antibody responses to pneumococcal vaccines and cryptococcal infection, animal models and genomic studies of the organisms and their hosts. The overarching view of our work is that the answer to resistance and susceptibility to disease with most organisms lies at the interface of host-microbe interaction. 

## What was your most significant scientific accomplishment?

So far, my most significant scientific accomplishments are studies that have challenged current dogma and suggested new paradigms. First, is the translational and experimental studies we have done that show antibody immunity is likely to contribute to resistance to cryptococcosis. We hope this will lead to a better understanding of susceptibility to cryptococcosis in HIV-infected and HIV-uninfected persons and new ways to anticipate, treat, and prevent disease. The other area is our discovery of a novel mechanism of antibody action against pneumococcus that works directly on the bacterium. This work has broadened our understanding of mechanisms of antibody immunity and opens new possibilities for the design of antibody based therapies.

